# Long-Term Vestibular Outcomes in Cochlear Implant Recipients

**DOI:** 10.3389/fneur.2021.686681

**Published:** 2021-08-11

**Authors:** Kasper Møller Boje Rasmussen, Niels West, Luchen Tian, Per Cayé-Thomasen

**Affiliations:** ^1^Department of Otorhinolaryngology Head and Neck Surgery and Audiology, Rigshospitalet, University Hospital of Copenhagen, Copenhagen, Denmark; ^2^Faculty of Health and Medical Sciences, University of Copenhagen, Copenhagen, Denmark

**Keywords:** cochlear implant, vestibular testing, dysfunction, vertigo, video head impulse test, cervical vestibular-evoked myogenic potentials, caloric irrigation test, dizziness handicap inventory

## Abstract

**Background:** Vestibular dysfunction is likely the most common complication to cochlear implantation (CI) and may, in rare cases, result in persistent severe vertigo. Literature on long-term vestibular outcomes is scarce.

**Objective:** This paper aims to evaluate vestibular dysfunction before and after cochlear implantation, the long-term vestibular outcomes, and follows up on previous findings of 35 consecutive adult cochlear implantations evaluated by a battery of vestibular tests.

**Methods:** A prospective observational longitudinal cohort study was conducted on 35 CI recipients implanted between 2018 and 2019; last follow-up was conducted in 2021. At the CI work-up (T0) and two postoperative follow-ups (T1 and T2), 4 and 14 months following implantation, respectively, all patients had their vestibular function evaluated. Evaluation with a vestibular test battery, involving video head impulse test (vHIT), cervical vestibular evoked myogenic potentials (cVEMP), caloric irrigation test, and dizziness handicap inventory (DHI), were performed at all evaluations.

**Results:** vHIT testing showed that 3 of 35 ears had abnormal vHIT gain preoperatively, which increased insignificantly to 4 of 35 at the last follow-up (*p* = 0.651). The mean gain in implanted ears decreased insignificantly from 0.93 to 0.89 (*p* = 0.164) from T0 to T2. Preoperatively, 3 CI ears had correction saccades, which increased to 11 at T2 (*p* = 0.017). Mean unilateral weakness increased from 19 to 40% from T0 to T2 (*p* < 0.005), and the total number of patients with either hypofunctioning or areflexic semicircular canals increased significantly from 7 to 17 (*p* < 0.005). Twenty-nine percent of CI ears showed cVEMP responses at T0, which decreased to 14% (*p* = 0.148) at T2. DHI total mean scores increased slightly from 10.9 to 12.8 from T0 to T1 and remained at 13.0 at T2 (*p* = 0.368). DHI scores worsened in 6 of 27 patients and improved in 4 of 27 subjects from T0 to T2.

**Conclusion:** This study reports significant deterioration in vestibular function 14 months after cochlear implantation, in a wide range of vestibular tests. vHIT, caloric irrigation, and cVEMP all measured an overall worsening of vestibular function at short-term postoperative follow-up. No significant deterioration or improvement was measured at the last postoperative follow-up; thus, vestibular outcomes reached a plateau. Despite vestibular dysfunction, most of the patients report less or unchanged vestibular symptoms.

## Introduction

Cochlear implantation (CI) is regarded as a safe and minimally invasive procedure due to its low prevalence of severe complications (<2%) (1). However, because of the proximity to the vestibular organs, there is a risk of mechanical damage to the labyrinth, saccule, or the horizontal semicircular canal presumably caused by cochleostomy and insertion of the implant electrode (2). Vestibular dysfunction is likely the most common complication to CI. Vertigo or disequilibrium accounts for 25–30% of complications and is usually transient and presents as mild to moderate postoperative dizziness or imbalance (1, 3–5). Some CI recipients experience prolonged and severe vertigo affecting daily and social activities and report lower quality of life (6, 7). These severe cases of postimplantation vertigo may in part be explained by preoperative vestibular dysfunction as a result of underlying inner ear pathology, leading to bilateral vestibular areflexia after implantation (8). It is of utmost importance to identify these patients before implantation, as patients with severe dizziness handicap may be at risk of social isolation, anxiety, and depression and falls and injuries.

Vestibular evaluation may be performed before CI potentially to reduce the risk of permanent vestibular dysfunction (9). No standard protocol for vestibular testing exists; however, the following tests are the most widely applied tests, but usually not represented all together (10): The video Head Impulse Test (vHIT) measures the vestibulo-ocular reflex (VOR) originating from the three semi-circular canals (horizontal, lateral, and posterior) (11), caloric irrigation tests the horizontal semi-circular canal and the inferior branch of the vestibular nerve, and cervical vestibular evoked myogenic potentials (cVEMP) tests the saccule and the inferior branch of the vestibular nerve (2); and the dizziness handicap inventory (DHI), a commonly used assessment tool for evaluation of quality of life in vestibular disorders (12). Previous studies apply various test strategies often resulting in contradictive findings (13–15).

Cochlear implantation can lead to vestibular impairment (16–20), and most frequently, the cVEMP and VOR are affected (13, 15, 21). Although vHIT combined with corrective saccades is a sensitive measure for detecting the impaired ear prior to implantation (16, 22), it has also been suggested that this test may be the least affected measure following implantation (10, 23). Excluding one of these tests may lead to an underestimation of vestibular affection after cochlear implantation (1, 10). Poor correlations between DHI scores and objectively measured outcomes are found in most studies, and in some studies, subjective improvement in self-perceived vertigo is indicated, despite objective vestibular deterioration (3, 13, 17, 24–26). Most of these studies urge for further research in long-term outcomes (27, 28).

We aim to evaluate long-term vestibular dysfunction after cochlear implantation, using a broad test battery including four vestibular test measures used in the clinical setting at our tertiary CI censer. Currently, all patients are subjected to vestibular examination, to guide our clinicians in the choice of treatment. We will report raw data on a prospective patient cohort and examine differences in vestibular test results at the short- and long-term postoperative follow-ups. The study therefore aims to provide important evidence on vestibular dysfunction after cochlear implantation and may show possible correlations between the subjective and objective outcome measures.

## Materials and Methods

### Study Design

The study design was prospective and observational and included participants who met the following inclusion criteria: adult (>18 years) first time CI recipients having bilateral moderate to profound sensorineural hearing loss (SNHL) eligible for CI. Individuals who were unfit for participation due to blindness, language barriers, patient reluctancy, or poor cooperation were excluded. Recruitment took place between February 2018 and April 2019, and the last follow-ups were carried out in February 2021.

The round window surgical procedure was applied throughout the study and performed by a team of senior CI surgeons. Each participant was assessed thrice: before implantation (in this study, referred to as T0), participants underwent vHIT, caloric irrigation, and cVEMP and completed the DHI. This test battery was repeated at two postoperative follow-ups at ~4 and 14 months after implantation, respectively (referred to as T1 and T2). All tests at all time points were performed by the same vestibular pathologist. The tests were performed with the CI turned on. No routine physiotherapy was performed postimplantation. However, patients experiencing marked dizziness were offered vestibular rehabilitation.

### Video Head Impulse Test

Impulsive testing of the lateral semicircular canals was measured using the Eyebeams vHIT system (Interacoustics, Middelfart, Denmark) with lightweight vHIT goggles, to test bilateral vestibulo-ocular reflexes (VOR) (29). Prior to testing, calibration of the equipment was performed according to standard recommendations. Patients were instructed to sit in an upright position and fixate on a visual target in front of them. The vestibular pathologist, standing behind the patient, generated the head impulses by moving the patient's head abruptly and unpredictably in the horizontal plane ~10–20° to each side with a range of peak head velocity between 150 and 300°/s. Any impulses outside this range were rejected by the software. Peak head velocities pre- and postimplantation were comparable. Head impulses were repeated 5–10 times each side until a satisfying result was recorded. The implant did not affect the placement of the vHIT strap or delivery method. Gain is calculated by the ratio of head velocity to eye velocity. vHIT gain results were considered abnormal if the gain was equal to or below 0.7. Gain asymmetry ratio (AR) was calculated by

(1) $$\begin{array}{r} GA=\left[\frac{G_{CL}-G_{CI}}{G_{CL}+G_{CI}}\right]\times 100 \end{array}$$

where *G*_*CL*_ denotes contralateral ear mean gain, and *G*_*CI*_ denotes cochlear implant ear mean gain (30). Gain AR >8.5% is considered abnormal.

Bilateral vHIT gains were measured at 40, 60, and 80 ms; however, only 60 ms was analyzed. Catch-up saccades were recorded and were considered abnormal in the presence of consistent overt or covert correction saccades, which depended on the amplitude of the saccade as a qualitative measure. Saccades with corresponding normal vHIT gain values were also considered abnormal.

### Caloric Irrigation

Low frequency testing of the lateral semicircular canals by caloric irrigation test was performed at standard caloric temperatures (30 and 44°C) by water stimulation (Aqua Stim, Interacoustics) and measured using videonystagmography (VNG). To improve patient communication, warm irrigations were performed before cold irrigations, and the worst hearing ear was tested first. Duration of the irrigations was 30 s, followed by a 60-s pause allowing eye monitoring. A 5-min pause between each irrigation was standard. Patients were denied visual input and performed alerting tasks to reduce central suppression during caloric testing. Slow phase velocity (SPV) was considered, and values below 25°/s were considered abnormal. Any outliers were excluded by the software. Unilateral weakness, a measure of afferent vestibular function loss, defined as partial (>25%, e.g., canal paresis) or complete (100%, e.g., unilateral areflexia), was calculated by the software using Jongkees formula.

### Cervical Vestibular Evoked Myogenic Potential

Otolithic function was measured using the vestibulospinal reflex elicited in response to cVEMP. Prior to cVEMP testing, the test equipment (Eclipse, Interacoustics) controls for EMG activation. In-ear air-conducted sound stimuli (100 dBnHL tone bursts at 500 Hz) (31) were used, and the electrode monitoring the elicited myogenic response was placed on the sternocleidomastoid muscle. cVEMP responses with both P1 and N1 present were considered [dichotomous outcomes (+/–)]. We did not report on the cVEMP asymmetry ratio because of the high variability of results reported in the literature (23).

### Dizziness Handicap Inventory

Preoperatively (T0) and at each of the two follow-ups (T1 and T2), patients completed the 25-item DHI, answering questions regarding perceived severity of vertigo and effects on quality of life (12). Patients rated each item with “yes,” “sometimes,” or “no,” corresponding to 4, 2, and 0 points, respectively. The total DHI scores range from 0 (no self-reported symptoms) to 100 (severe self-reported symptoms). A score of 0–15 points corresponds to no handicap, 16–34 points corresponds to mild handicap, 35–52 points corresponds to moderate handicap, and 53–100 corresponds to severe handicap (20). Validity evidence of the DHI shows that an 11-point difference is considered significantly different between repeated measures (12).

### Statistical Analysis

Statistical processing was carried out in SPSS (32), and graphs were processed with GraphPad Prism (33). Descriptive data were evaluated by number, mean, 95% confidence intervals, and percentage. Distribution normality was tested using boxplots and QQ plots. vHIT gain and SPV were normally distributed; vHIT gain AR, UW, and DHI scores were non-normally distributed. Saccades and cVEMP results are dichotomous data. Parametric data are analyzed using repeated measures linear mixed models with an unstructured covariance structure. Friedman test was applied on non-parametric data and Cochrane's Q test on dichotomous data, both designed to analyze repeated measures. Analyses were carried out to determine whether there was any statistically significant difference between preimplantation (T0) and postimplantation (T1 and T2). Spearman (r) correlation analysis was conducted when the significance of the relationships was tested. The significance level was a two-tailed *p* < 0.05.

## Results

Forty-three patients were initially included. Three patients later withdrew because of reluctancy due to coronavirus disease 2019 (COVID-19). Another five patients were excluded: one patient was explanted due to late-onset device infection, one moved abroad, one had deceased, and two had comorbidity that excluded them from the study. All participants received unilateral CI; two of these were consequentially implanted on the contralateral side. Data from both unilateral and bilateral CI recipients were included. Table 1 shows demographic characteristics, Table 2 reports raw data for the 35 patients, and Table 3 summarizes the data.

**Table 1:** Demographic characteristics of the 35 study patients.

Age	26–85 years (mean, 59)
Gender	16 female (46%), 19 male (54%)
Implanted side	17 left, 16 right, 2 bilateral
Type of implant	1 (3%) Advanced Bionics HiRes Ultra 3D SlimJ
	1 (3%) Advanced Bionics HiRes90K Midscale
	1 (3%) Advanced Bionics ULTRA 3D Midscale
	1 (3%) MEDel Flex 28 Synchrony
	21 (60%) Nucleus Cochlear CI522
	6 (17%) Nucleus Cochlear CI622
	4 (11%) Oticon Medical Zti EVO
**Days after implantation**	
T1	122 days, 69–222 (mean, range)
T2	406 days, 265–532 (mean, range)

**Table 2:** Complete raw data set on all 35 patients.

**ID**	**Hearing loss aetiology**	**vHIT gain**						**vHIT gain** **asymmetry ratio (%)**			**Saccades**		**cVEMP**		**SPV total**			**UW**			**DHI**		
		**CI ear**			**Non-CI ear**						**CI ear**	**Non-CI ear**	**CI ear**	**Non-CI ear**									
		T0	T1	T2	T0	T1	T2	T0	T1	T2	T0/T1/T2	T0/T1/T2	T0/T1/T2	T0/T1/T2	T0	T1	T2	T0	T1	T2	T0	T1	T2
1	Ménière's disease	0.65	0.73	0.73	0.87	0.88	0.88	14.5	9.3	9.3	ov/0/ov	0/ov/0	NA/–/–	NA/–/–	NA	NA	46.6	NA	6	6	12	32	32
2	Otitis media	1.10	1.01	1.06	1.00	1.06	1.08	4.8	2.4	0.9	0/0/0	0/0/0	–/–/–	–/–/–	244.10	9.3	92.6	7	46	58	36	26	30
3	Otosclerosis	0.84	0.39	0.36	1.00	0.67	0.67	8.7	26.4	30.1	0/0/ov + cov	0/ov + cov/ov + cov	–/–/–	–/–/–	23.30	39.2	17.6	14	55	100	28	20	NA
4	Congenital (unknown aetiology)	0.86	1.00	0.86	0.94	0.95	0.95	4.4	2.6	5.0	0/0/0	0/0/0	+/+/+	+/+/+	121.10	108.2	109.6	22	21	24	0	0	0
5	Unknown	0.79	0.79	0.85	1.04	0.74	0.73	13.7	3.3	7.6	0/0/0	0/0/0	–/–/–	–/–/–	161.2	121.3	129.5	4	20	10	0	0	0
6	Unknown	1.13	0.81	0.76	0.94	1.05	0.89	9.2	12.9	7.9	0/0/ov	ov/ov/ov	–/–/–	–/–/–	82.0	27.4	29.5	6	60	76	0	38	52
7	Late-onset progressive hereditary	1.23	0.97	1.41	1.14	1.12	1.29	3.8	7.2	4.4	0/0/0	0/0/0	–/+/+	–/+/+	134.3	108.2	121.5	19	7	12	0	6	14
8	Superficial siderosis	0.55	0.45	0.43	0.57	0.65	0.41	1.8	18.2	2.4	ov/ov/ov	ov/ov/0	–/–/–	–/–/–	23.4	18.6	11.2	43	23	34	60	64	72
9	Late-onset progressive hereditary	0.93	0.77	0.73	0.87	0.96	0.88	3.3	11.0	9.3	0/0/0	0/0/0	–/–/–	–/–/–	84.8	61.8	78.0	40	30	23	0	0	0
10	Hereditary congenital	0.73	0.82	0.97	0.80	1.11	1.07	4.6	15.0	4.9	0/ov/0	0/0/0	–/–/–	–/–/–	95.7	69.2	69.9	19	100	83	0	0	0
11	Unknown	1.31	0.89	1.09	1.02	0.91	1.08	12.4	1.1	0.5	0/0/0	0/0/0	–/–/–	–/–/–	NA	65.3	50.0	NA	86	22	0	0	0
12	Unknown	1.05	1.02	1.10	1.14	1.07	1.02	4.1	2.4	3.8	0/0/0	0/0/0	–/–/–	–/–/–	118.5	92.5	98.4	14	29	22	0	6	0
13	Usher syndrome	0.95	0.81	0.93	0.89	0.88	0.94	3.3	4.1	0.5	0/0/0	0/0/0	–/–/–	–/–/–	116.0	147.8	143.9	30	47	6	0	0	0
14	Late-onset progressive hereditary	0.88	0.69	0.88	0.96	0.75	0.88	4.3	4.2	0.0	ov/ov/ov	ov/ov/ov	+/+/–	+/+/–	22.5	23.0	18.8	25	2	42	10	20	10
15	Late-onset progressive hereditary	1.10	1.03	1.14	0.97	0.89	0.97	6.3	7.3	8.1	0/ov/0	ov/ov/ov	–/–/–	–/–/–	92.8	71.1	60.0	65	74	57	80	56	34
16	Unknown	0.73	0.25	0.21	0.81	0.88	0.72	5.2	55.8	54.8	0/ov/ov	0/0/ov	+/–/–	+/+/–	40.5	13.1	19.8	20	100	100	0	0	NA
17	Late-onset progressive hereditary	0.99	1.01	1.04	0.96	0.98	1.08	1.5	1.5	1.9	0/0/cov	0/0/cov	–/–//NA	–/+/NA	84.7	52.4	35.8	9	24	5	0	0	0
20	Unknown	1.00	1.04	0.84	0.93	1.15	0.87	3.6	5.0	1.8	0/0/cov	0/0/cov	+/–/NA	+/–/NA	116.0	91.9	85.9	8	32	69	0	0	NA
22	Unknown	1.09	1.06	1.09	1.12	1.06	1.09	1.4	0.0	0.0	0/0/0	0/0/0	–/–/NA	–/+/NA	70.6	45.0	43.5	27	16	11	0	4	NA
23	Unknown	0.92	0.84	0.88	0.85	0.90	0.81	4.0	3.4	4.1	0/0/ov	0/0/ov	–/–/+	–/+/+	87.3	71.2	64.5	51	75	76	30	52	NA
25	Congenital (unknown aetiology)	0.80	0.76	0.90	0.76	0.86	0.68	2.6	6.2	13.9	0/0/0	0/0/ov	–/–/–	–/–/–	50.7	24.2	29.6	18	20	28	0	6	12
27	Pendred syndrome	0.96	1.10	1.04	1.04	1.18	1.04	4.0	3.5	0.0	0/0/cov	0/0/cov	+/+/+	+/+/+	48.8	62.0	51.0	1	23	25	0	0	0
28	Hereditary congenital	0.74	0.76	0.93	0.67	0.76	0.81	5.0	0.0	6.9	0/0/cov	0/0/ov	–/–/–	–/–/–	NA	NA	NA	NA	NA	NA	0	0	0
29	Unknown	0.99	1.03	1.21	1.06	1.07	1.48	3.4	1.9	10.0	0/0/0	0/0/0	–/–/+	–/–/+	NA	60.0	78.7	NA	29	10	0	56	46
30	Late-onset progressive hereditary	1.04	0.95	0.71	1.09	0.92	1.08	2.3	1.6	20.7	0/ov/0	0/0/0	–/–/–	–/–/–	143.5	96.6	102.5	6	28	28	0	0	0
31	Unknown	1.19	0.85	0.93	1.10	1.05	0.96	3.9	10.5	1.6	0/0/0	0/0/0	+/–/–	+/–/+	28.6	26.3	21.1	15	16	14	0	0	0
32	Unknown	0.89	1.05	0.87	1.06	1.06	0.84	8.7	0.5	1.8	0/0/0	0/0/0	+/–/NA	+/+/NA	57.5	74.2	67.1	5	22	13	0	0	0
33	Otosclerosis	0.80	0.95	0.82	0.75	1.07	0.73	3.2	5.9	5.8	0/0/0	0/0/ov	+/–/–	+/+/+	60.2	53.2	50.9	18	14	16	0	0	0
34	Pneumococcal meningitis	0.50	0.90	0.78	0.73	1.00	0.88	18.7	5.3	6.0	0/0/0	0/0/ov	–/–/–	–/–/–	57.9	32.2	19.9	19	12	27	38	28	44
36	Late-onset progressive hereditary	1.29	1.22	1.16	1.41	1.40	1.26	4.4	6.9	4.1	0/0/0	0/0/0	+/–/–	+/+/–	113.4	58.3	74.6	28	71	89	12	5	0
39	Unknown	1.06	0.91	0.75	0.92	0.97	0.78	7.1	3.2	2.0	0/0/0	0/0/0	–/–/–	–/–/–	137.9	70.1	50.4	16	36	0	46	0	0
40	Unknown	0.82	0.93	0.90	0.91	1.02	0.94	5.2	4.6	2.2	0/0/0	0/0/0	–/–/–	–/–/–	56.5	40.7	35.4	4	7	73	0	2	6
41	Usher syndrome	1.03	1.10	0.65	1.10	1.07	0.78	3.3	1.4	9.1	0/0/0	0/0/0	–/–/NA	–/–/NA	128.8	88.2	52.9	7	29	27	0	0	NA
42	Late-onset progressive hereditary	0.84	0.91	0.91	0.87	0.98	0.97	1.8	3.7	3.2	0/0/0	0/0/0	+/+/–	+/+/+	81.0	52.7	54.2	21	4	14	0	0	NA
43	Hereditary congenital	0.91	0.63	1.19	0.90	0.77	1.25	0.6	10.0	2.5	0/0/0	0/ov/0	–/–/–	–/–/+	34.5	17.3	15.1	3	100	64	0	28	NA

*CI, cochlear implant; cov, covert; ov, overt; cVEMP, cervical vestibular evoked myogenic potential; DHI, dizziness handicap inventory; NA, not available; T0, baseline before implantation; T1, first postoperative follow-up; T2, second postoperative follow-up; SPV, slow phase velocity; UW, unilateral weakness; VHIT, video head impulse test; +, present response; −, absent response*.

**Table 3:** Summary data of vestibular test battery results at baseline before implantation (T0), first postoperative follow-up (T1), and second postoperative follow-up (T2).

	**T0**	**T1**	**T2**
**Video head impulse test**			
Implanted ear, mean gain (95% CI)	0.93 (0.87–1.00)	0.87 (0.80–0.94)	0.89 (0.81–0.97)
Non-implanted ear, mean gain (95% CI)	0.95 (0.89–1.00)	0.97 (0.91–1.02)	0.94 (0.87–1.01)
vHIT gain asymmetry ratio (%), mean (95% CI)	5.4 (4.0–6.8)	7.4 (3.9–10.8)	7.1 (3.5–10.6)
Abnormal vHIT gain on implanted ear, n (%)	3 (9)	5 (14)	4 (11)
Abnormal vHIT gain on non-implanted ear, n (%)	2 (6)	2 (6)	3 (9)
Abnormal vHIT gain asymmetry ratio, n (%)	7 (20)	9 (26)	8 (23)
Correction saccades present on implanted ears, n (%)	3 (9)	6 (17)	11 (31)
Correction saccades present on non-implanted ears, n (%)	4 (11)	7 (20)	12 (34)
**Caloric irrigation test**			
Slow phase velocity (SPV, °/s), mean (95% CI)	87.7 (69.8–105.6)	60.2 (47.5–73.0)	59.8 (46.4–73.2)
Unilateral weakness (%), mean (95% CI)	19 (13–24)	37 (26–47)	40 (28–51)
Hypofunction, n (%)	7 (20)	15 (43)	15 (43)
Areflexia, n (%)	0	3 (9)	2 (6)
**Cervical vestibular evoked myogenic potentials**			
cVEMP present on implanted ears, n (%)	10 (29)	6 (17)	5 (14)
cVEMP present on non-implanted ears, n (%)	10 (29)	12 (34)	9 (26)
**Dizziness handicap inventory**			
Total score, mean (95% CI)	10.9 (2.4–19.4)	12.8 (4.9–20.7)	13.0 (4.9–21.1)
No handicap (0–15), n (%)	28 (80)	24 (69)	20 (74)
Mild handicap (16–34), n (%)	2 (6)	6 (17)	3 (11)
Moderate handicap (35–52), n (%)	3 (9)	2 (6)	3 (11)
Severe handicap (53−100), n (%)	2 (6)	3 (9)	1 (4)

**Figure 1:**
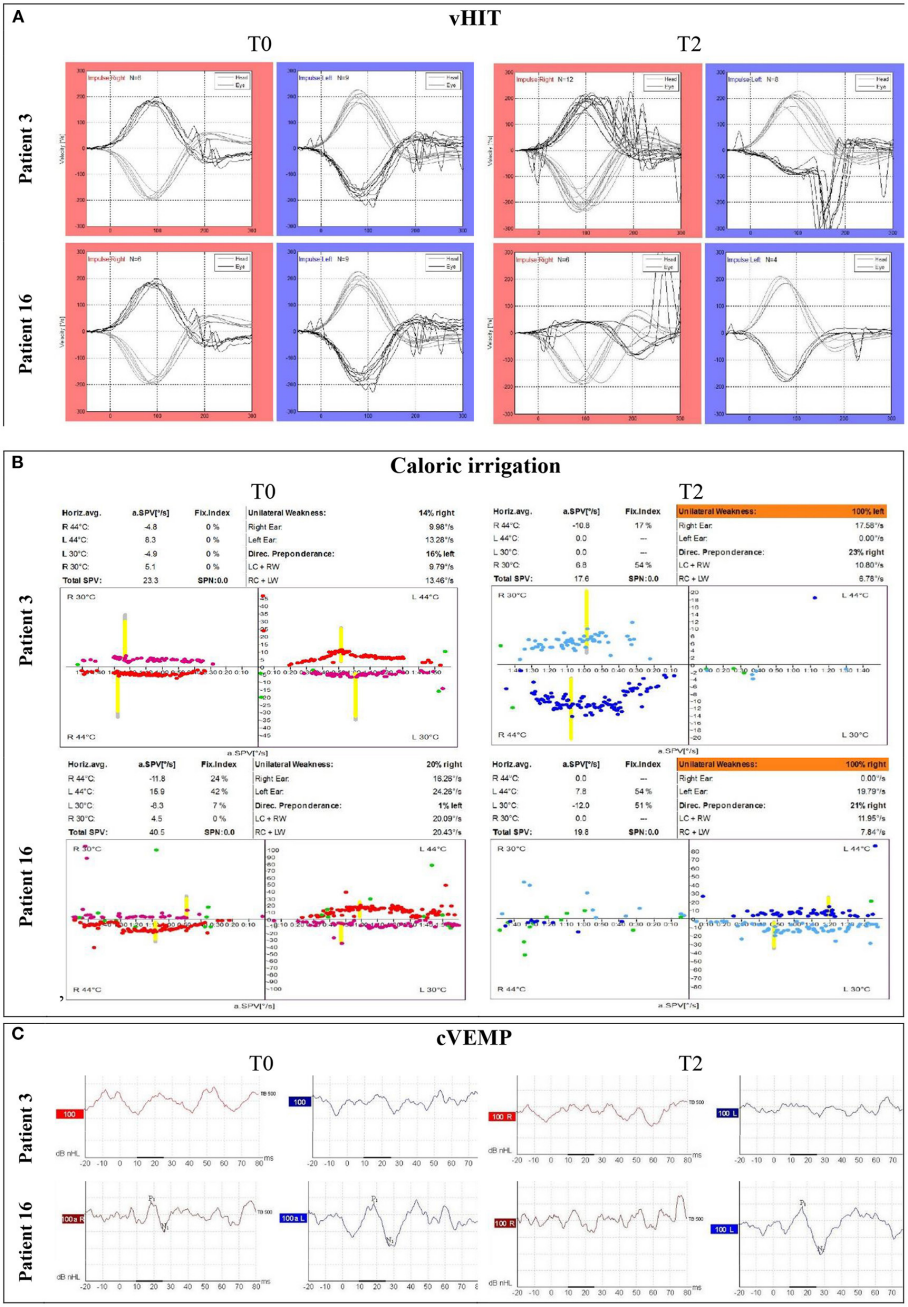
Preoperative and postoperative raw data for patients 3 and 16 at T0 and T2, respectively. **(A)** vHIT tracings, **(B)** raw caloric irrigation test data including total SPV and UW, and **(C)** cVEMP tracings.

Patients 3 and 16 (raw data represented in Figure 1 and highlighted in Table 1) demonstrated clearly deteriorating vestibular function from T0 to T2. At baseline, they presented normal vHIT gains, normal vHIT gain asymmetry ratio, no saccades, and unilateral weakness lower than 25%. Patient 16 showed a positive cVEMP response at T0; however, patient 3 did not. At T2, both patients had a vHIT gain drop below 0.70 and vHIT gain asymmetry ratio above 8.5%. Both had developed corrective saccades, none of them had positive cVEMP responses at T2, and both had unilateral canal paresis (UW = 100%). Interestingly, patient 16 did not report any symptoms of vertigo in the DHI at T0 and T1, and patient 3 scored 28 at T0, which dropped to 20 at T1 (mild handicap). None of the patients answered DHI at T2. On the contrary, some patients (e.g., ID 27, 29, and 34) show clear improvement in vHIT. However, both patients 29 and 34 report moderate handicaps in DHI.

### vHIT Results

**Figure 2:**
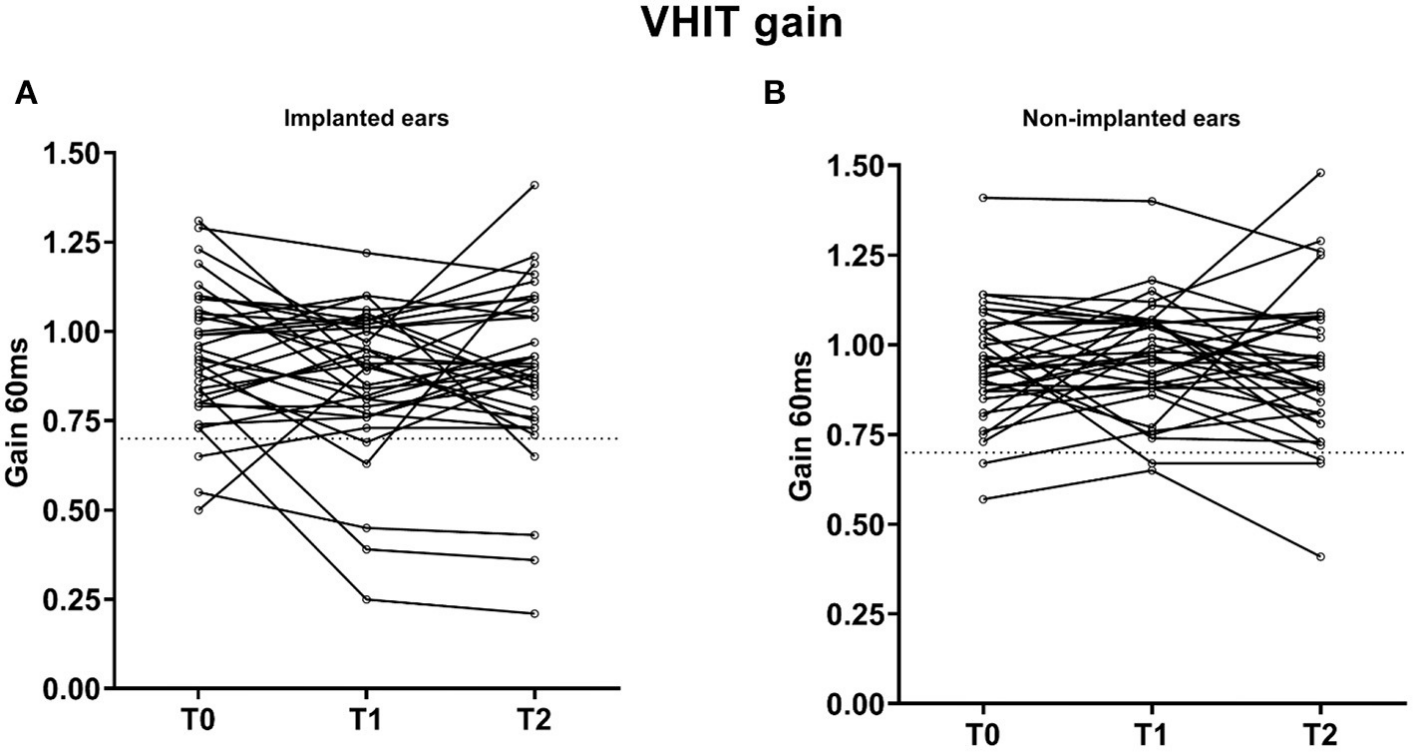
Individual changes in vHIT gains (*n* = 35) on **(A)** implanted and **(B)** non-implanted ears, respectively. Patients 3, 8, and 16 stand out with the lowest vHIT gain values. ms, milliseconds; T0, baseline before implantation; T1, first postoperative follow-up; T2, second postoperative follow-up; vHIT, video head impulse test. Observations below the dotted line are considered abnormal (vHIT = 0.7).

**Figure 3:**
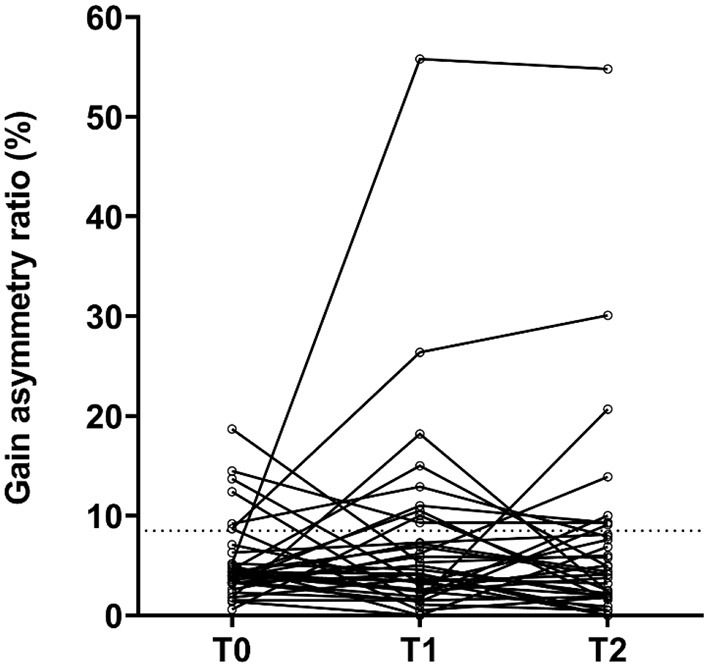
Individual changes in vHIT gain asymmetry ratio (AR, %) (*n* = 35). T0, baseline before implantation; T1, first postoperative follow-up; T2, second postoperative follow-up. Observations above the dotted line are considered abnormal (gain AR = 8.5%).

On implanted ears, a total of three (9%), five (14%), and four (11%) patients had abnormal vHIT gain values at T0, T1, and T2, respectively (Table 3 and Figure 1). There was no significant difference between T0, T1, and T2 (*p* = 0.164). On implanted ears, three patients (ID 3, 16, and 41) changed from preoperatively normal gain values to abnormal T2 gain values on their implanted ears. As summarized in Table 3, three patients (ID 1, 8, and 34) presented abnormal vHIT gain values on the implanted ear. At T2, four patients (ID 3, 8, 16, and 41) had abnormal vHIT gain values, so three patients had deteriorated at T2. Patients 3, 8, and 16 stands out in Figure 2 by having the lowest vHIT gain values. One patient (ID 8) had abnormal gain values in all tests in both ears. Eight patients had abnormal vHIT gain AR at T2 compared to the seven at T0—again patients 8 and 16 stood out. Mean vHIT gain asymmetry ratio did not change significantly (*p* = 0.917), but patients 3, 16, and 30 had a marked increase at T2 (Table 2 and Figure 2). Correction saccades occurred as reported in Table 3 and Figure 3. A significant increase in present correction saccades on both CI ears and contralateral ears was observed (*p* = 0.017 for both analyses).

### Caloric Irrigation Results

**Figure 4:**
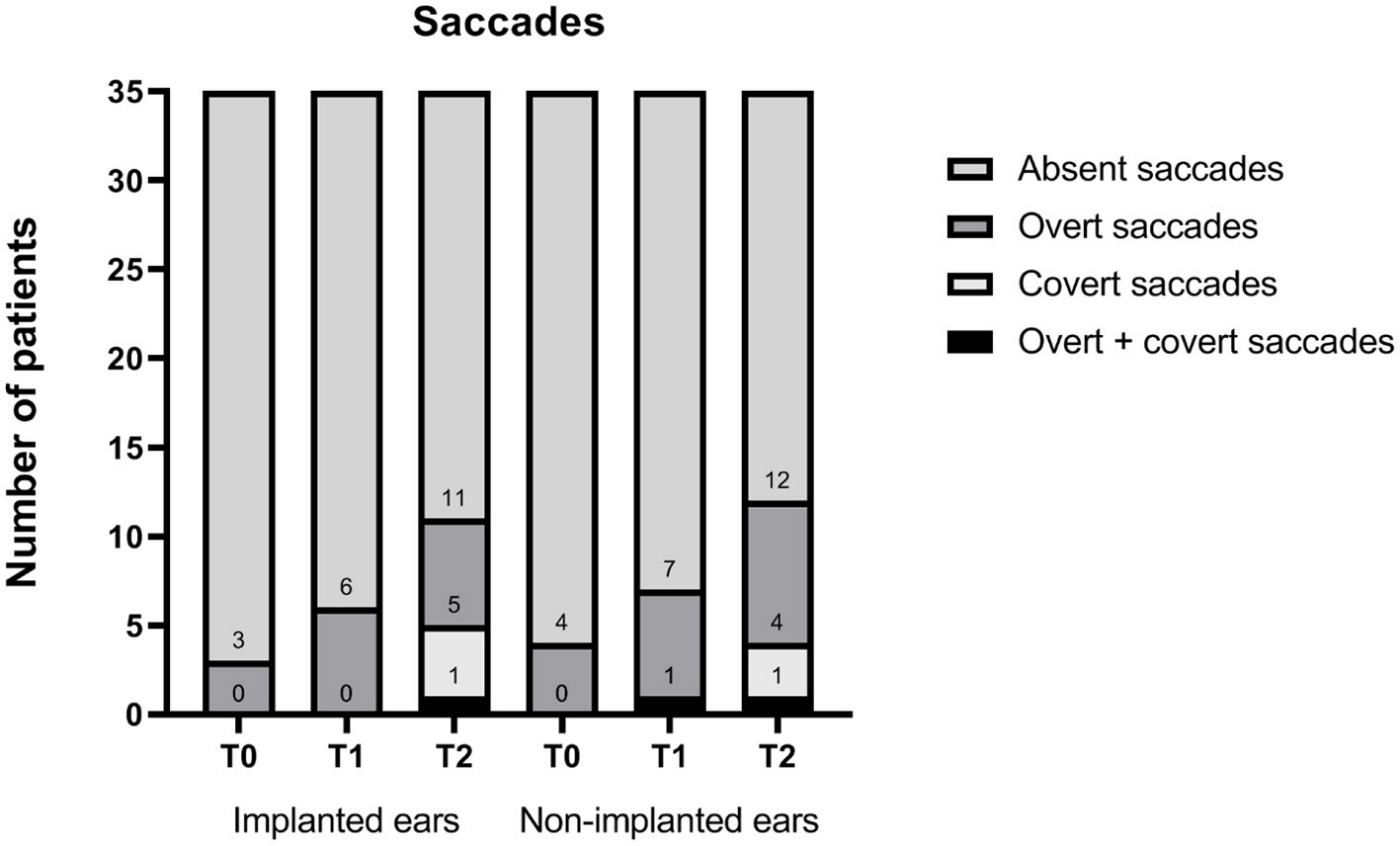
Pre- and postoperative saccades for implanted and non-implanted ears (*n* = 35). T0, baseline before implantation; T1, first postoperative follow-up; T2, second postoperative follow-up. Numbers indicate accumulated sums.

On implanted ears, the mean total SPV was 87.7°/s at T0, 60.2°/s at T1, and 59.8°/s at T2 (Table 3 and Figure 4). A significant decrease in SPV was observed from preimplantation to short-term follow-up (T1) (*p* < 0.005) and stayed low at T2 (*p* = 0.915).

At T0, the mean unilateral weakness was 19%. UW increased significantly to 37% at T1 and 40% at T2 (*p* < 0.005). Eleven patients (ID 2, 3, 6, 10, 16, 20, 27, 30, 36, 40, and 43) all had noticeable increases in UW. Seven patients (20%) had caloric hypofunction preimplantation, and none were areflexic. This number increased significantly to 18 patients at T1 and 17 patients at T2 (*p* < 0.005), and of those, 3 (9%) and 2 (6%) patients had areflexia, respectively. Caloric test data were incomplete for four patients.

### cVEMP Results

**Figure 5:**
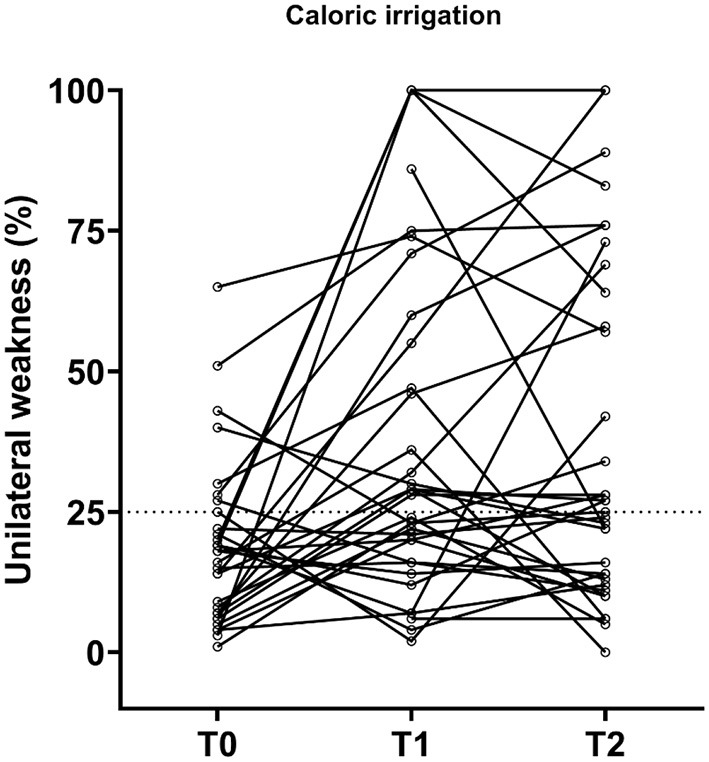
Individual changes in caloric function before implantation and at the postoperative follow-ups. T0, baseline before implantation; T1, first postoperative follow-up; T2, second postoperative follow-up. Observations above the dotted line represent vestibular hypofunction (UW = 25%); UW = 100% means areflexia.

At T0, 10 patients (29%) presented cVEMP responses on the implanted ears, and 10 (29%) patients presented cVEMP responses on non-implanted ears (Figure 5). At T2, five cVEMP responses were lost on the implanted ears, and one was lost at the non-implanted ears. No significant difference was observed on both ear when comparing all three time points (*p* = 0.148). Patients with present cVEMP responses at T2 had a mean vHIT gain of 1.05 (*n* = 5) compared with 0.86 (*n* = 30) in the group with absent cVEMP. The mean unilateral weakness was 29.5% (*n* = 5) in the group with present cVEMPs compared with 38.5% (*n* = 30) in the group with absent cVEMPs. cVEMP data were incomplete for six patients.

### DHI Results

**Figure 6:**
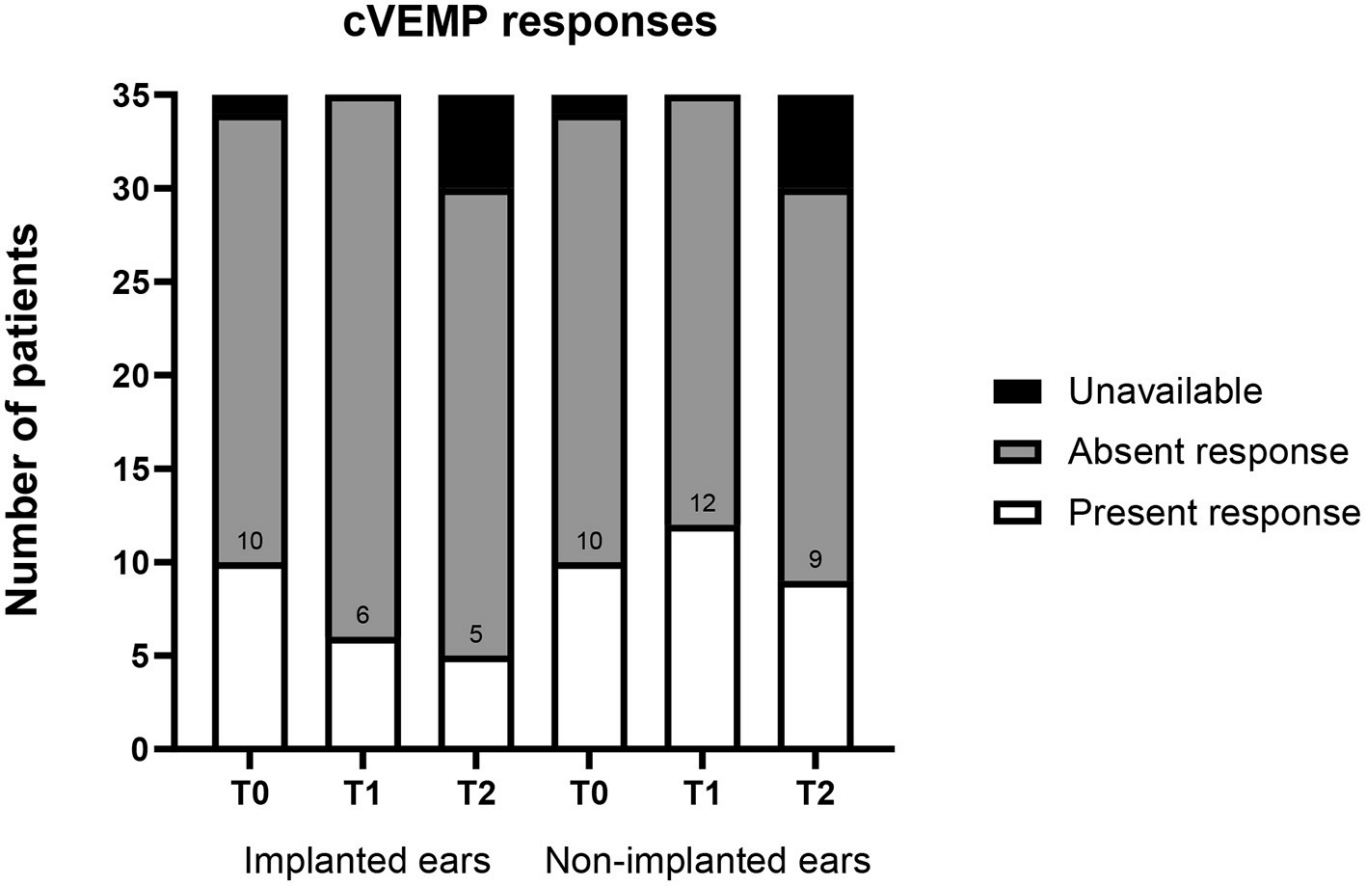
Pre- and postoperative cVEMP responses for implanted and non-implanted ears (*n* = 35). T0, baseline before implantation; T1, first postoperative follow-up; T2, second postoperative follow-up; cVEMP, cervical evoked myogenic potentials. Numbers indicate number of present responses.

DHI mean scores at T0, T1, and T2 were 10.9, 12.8, and 13.0, respectively (Table 3 and Figure 6). No significant differences in DHI totals were found pre- and postimplantation DHI scores (*p* = 0.368). At T2, 16 patients (36%) reported no symptoms (DHI = 0), but 2 patients (6%) improved and 7 patients (20%) worsened compared to their T0 value. Eight patients had incomplete DHI data at T2. At T2, no association were found between the DHI scores and caloric irrigation (r_s_ = 0.369; *p* = 0.084) and vHIT gain (r_s_ = −0.313; *p* = 0.259) on implanted ears. Most frequently, patients reported DHI total scores corresponding to no handicap. Two patients (ID 8 and 15) had severe handicaps at T0. As the only patient in the cohort, Patient 8 was still seriously affected by dizziness at T2.

**Figure 7:**
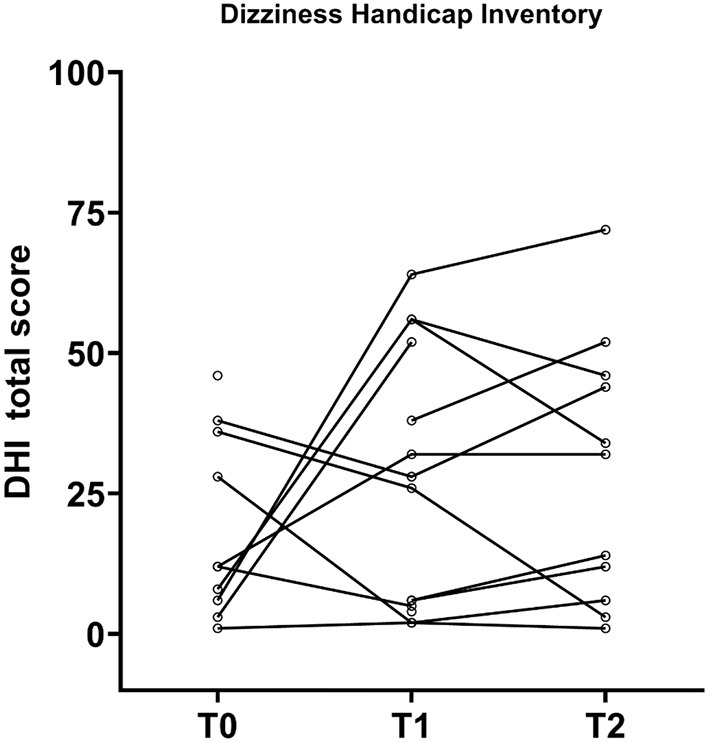
Patient-reported vestibular outcomes measured by the dizziness handicap inventory (DHI) before and after implantation (*n* = 35). No significant change in total score were reported between pre- and postimplantation. Eighteen patients with both pre- and postoperative DHI 0 are not visualized on the plot. T0, baseline before implantation; T1, first postoperative follow-up; T2, second postoperative follow-up.

## Discussion

We herein present follow-up data to a previously conducted study from our institution (3) investigating postoperative vestibular function in a patient cohort after cochlear implantation. In the present study, we investigated long-term vestibular outcomes of 35 cochlear implant recipients using a recognized set of vestibular tests and correlated the results from two consecutive postoperative follow-ups. This study adds evidence to the research field regarding vestibular dysfunction after cochlear implantation. We applied a comprehensive vestibular test battery on a medium-sized patient cohort and found that patients' vestibular function deteriorated 3–6 months after implantation and tended to stabilize ~14 months postimplantation. The present study observes no associations between objective vestibular testing and self-reported symptoms, and only a small group of patients report moderate to severe dizziness symptoms. In this study, we found no significant decrease in vHIT gain but a significant increase in number of corrective saccades. Unilateral weakness increased significantly, but no correlation between vHIT and caloric irrigation was observed. cVEMP responses were reduced although not significantly.

In summary, 9% showed preoperatively abnormal vHIT gains, 20% had hypofunctioning caloric responses, and 61% did not elicit cVEMP responses. Postoperatively at first follow-up, an overall deterioration in vestibular function was observed, as 14% of patients had abnormal vHIT gains, 52% had either caloric hypofunction including areflexia, and 83% had absent cVEMP responses. The results from the last postoperative follow-up showed a stabilization of vestibular dysfunction: 11% had abnormal vHIT gain, 49% had either hypofunctioning or areflexic caloric responses, and 86% had absent cVEMP responses. Despite detectable vestibular dysfunction, fewer patients from T0 to T2 exhibit moderate or severe handicaps. Thus, the DHI score remains virtually unchanged, suggesting that the symptom burden has plateaued. Even though vestibular function is preserved objectively, the patients may experience symptoms and vice versa. Therefore, the results show that the objective and subjective vestibular outcomes are inconsistent, which also has been stated by West et al. (3) and supported by other conducted studies in the field (23). In a systematic review including 27 studies, Ibrahim et al. showed great variability in vestibular test results but concluded that CI surgery can significantly affect caloric irrigation and cVEMP responses but not vHIT and DHI. The authors argued that the effect is clinically insignificant because DHI total scores was not affected by cochlear implantation (10). Other studies also reported diverse and often contradictory results (17, 19, 20). The current study shows that when we focus on individual patients, we may see both worsening and improving vestibular function. The causes of vestibular dysfunction after cochlear implantation have been attributed to various factors including direct surgical trauma, endolymphatic hydrops, and inflammatory reaction (34). These theories are supported by histopathological studies, which have revealed vestibular organ damage in post-mortem specimens (35, 36). Additionally, the vestibular deficits pre- and postimplantation may differ depending on the hearing impairment aetiology. For instance, patient 8 with superficial siderosis presented bilateral vestibular dysfunction, and the present test battery may be incapable of determining any meaningful difference pre- and postimplantation. On the other hand, it has been argued that vestibular function improves with recovering auditory function. Due to better auditory function, patients become increasingly socially and physically active, improving their postural function and well-being. According to Colin et al., this improvement in quality of life may improve the subjective feeling of balance (17). This could reinforce the idea proposed by Abouzayd et al. that we need to apply a case-by-case strategy based on the patient's symptoms and hearing impairment aetiology (23).

Systematic long-term vestibular assessment after cochlear implantation has been performed in two previous studies (27, 37), reporting caloric outcomes and vHIT/cVEMP outcomes, respectively. Another study performed the caloric test, posturography, and rotatory chair test 365 days postimplantation but failed to provide quantitative results from the follow-up, making comparisons difficult (38). Buchman et al. reported that 29% of their cohort had a substantial reduction in the slow velocity VOR, as measured by the caloric test (27). In the recent paper from our institution, 45 individuals were retrospectively evaluated long term after cochlear implantation. It was found that the high velocity VOR function as measured by vHIT gain was preserved, but a tendency to demonstrate vHIT saccades on implanted ears was observed. Furthermore, cVEMP potentials were significantly reduced (21).

We found a discrepancy in self-perceived symptoms of vertigo and objective test findings, and one explanation may be that each test is unsuccessful in determining vestibular deficiency. Second, central compensation may alleviate symptoms of vertigo, while objective tests still detect vestibular dysfunction (25). Third, the findings may indicate that only subparts of the vestibular organs are evaluated, so the test battery is incapable of analyzing the full complexity of the vestibular apparatus. To our knowledge, no recent studies have examined all five vestibular organs. In this study, we focused on the lateral semicircular canal, tested with vHIT and caloric irrigation test, and sacculus, tested with cVEMP responses, and found some degree of vestibular deterioration. Although we consider this comprehensive test battery a strength to the study, not all vestibular end organs are studied. The vestibular test battery could also encompass impulsive testing (vHIT) of the RALP (right anterior and left posterior semicircular canals) and LARP (left anterior and right posterior semicircular canals), referred to as vertical vHIT. In addition, ocular VEMP (oVEMP) data were not part of the evaluation. Imai et al. previously demonstrated that oVEMP is a useful measure of utricular function (15). Thus, the omission of vertical vHIT and oVEMP may contribute to the outcome mismatch observed between subjective and objective vestibular evaluations. As we did not examine the remaining parts of the vestibular apparatus (i.e., anterior and posterior semicircular canals and utriculus), an existing and possibly enhanced association between objective vestibular test results may not have been appointed.

We did not observe a correlation between self-reported symptoms and objectively measured vestibular dysfunction, and it may be argued that psychological factors, such as anxiety and depression, contribute to the disagreement. Our study did not investigate development of anxiety and depression, and this may be a focus for future studies. Another study limitation includes patient loss to follow-up. There is no indication that patients dropped out due to severe vertigo; the reason was most likely due to the COVID-19 pandemic. Furthermore, the absolute follow-up may represent a limitation, as 14 months may be insufficient to determine the final vestibular function. Future studies should address whether vestibular function normalizes with longer postimplant follow-up time or if vestibular function has already reached a nadir 14 months following implantation. Age and aetiology of SNHL may affect vestibular function. Therefore, future studies are also needed to investigate whether age and aetiology of SNHL play a role in the end vestibular function or if the deterioration from 4 to 14 months merely is a result from an age-related dysfunction.

## Conclusion

We present a prospective observational study of long-term subjective and objective vestibular outcomes in 35 cochlear implant recipients. Our study demonstrates that cochlear implantation can worsen vestibular function and that long-term effects tend to plateau rather than deteriorate vestibular function. vHIT, caloric irrigation, and cVEMP all measured an overall worsening of vestibular function at long-term postoperative follow-up. Despite vestibular dysfunction, a large proportion of patients report less or unchanged vestibular symptoms. Pre- and post-implantation vestibular evaluations can give short- and long-term prognostic information, and guide implantation side selection, treatment, and vestibular rehabilitation.

## Data Availability Statement

The raw data supporting the conclusions of this article will be made available by the authors, without undue reservation.

## Ethics Statement

The studies involving human participants were reviewed and approved by The Danish National Committee on Health Research Ethics (H-17034918) and The Danish Data Protection Agency (RH-2017-308). The patients provided their written informed consent to participate in this study.

## Author Contributions

KR: drafting of the manuscript. NW and PC-T: study design. All authors revision of the manuscript, final approval, and final agreement.

## Conflict of Interest

The authors declare that the research was conducted in the absence of any commercial or financial relationships that could be construed as a potential conflict of interest.

## Publisher's Note

All claims expressed in this article are solely those of the authors and do not necessarily represent those of their affiliated organizations, or those of the publisher, the editors and the reviewers. Any product that may be evaluated in this article, or claim that may be made by its manufacturer, is not guaranteed or endorsed by the publisher.
